# Bikini-line Hiatal Hernia Repair (BLHHR) During Sleeve Gastrectomy

**DOI:** 10.1007/s11695-023-06881-z

**Published:** 2023-10-20

**Authors:** Tamer N Abdelbaki

**Affiliations:** General Surgery Department, Alexandria University Faculty of Medicine, Midan Khartoum, Alexandria, Egypt

**Keywords:** Bikini line, Hiatal hernia, Sleeve gastrectomy, Ergonomics, Aesthetic benefits

## Abstract

**Background:**

To preserve the aesthetic benefits achieved with Bikini line sleeve gastrectomy (BLSG), we have devised a novel approach for simultaneous hiatal hernia repair (HHR), known as bikini-line hiatal hernia repair (BLHHR). This manuscript presents our initial experience with BLHHR and assesses its feasibility and outcomes.

**Methods:**

A prospective preliminary study was conducted on patients who underwent BLHHR between September 2020 and October 2022. Patient demographics, preoperative assessments, operative details, postoperative outcomes, and aesthetic evaluations were recorded. Feasibility and safety were assessed.

**Results:**

Among 891 BLSG patients, 89 (9.9%) underwent BLHHR. The mean distances between the xiphoid process and the umbilicus, symphysis pubis, and anterior superior iliac spine (ASIS) were 28.8 ± 2.2, 33.9 ± 3.1, and 31.2 ± 1.8 cm, respectively. Optimal visualization and accessibility of the gastroesophageal junction (GEJ) were achieved without compromising HHR repair or sleeve gastrectomy. The mean operative time was 76.5 ± 11 min, longer than the 58 ± 10 min required for BLSG alone. Patient scar satisfaction ranged from 87.5 to 97.9%, and the mean pain score was 2.9 ± 0.8. No major complications were reported. At 6 months, %EWL (percentage of excess weight loss) was 53.3 ± 13.7%, GERD (gastroesophageal reflux disease) remission was achieved in 62.8% of patients and comorbidities were improved.

**Conclusion:**

BLHHR was potentially feasible and safe. Outcomes related to patient scar satisfaction, weight loss, improvement of associated comorbidities, and GERD symptoms were not compromised. The aesthetic benefits achieved by BLSG were maintained.

**Graphical Abstract:**

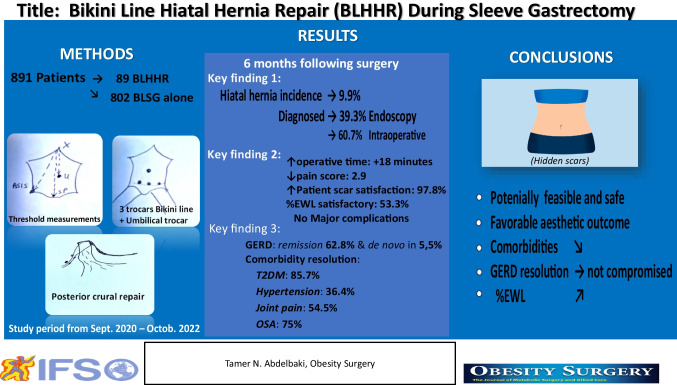

## Introduction

Laparoscopic sleeve gastrectomy (LSG) is currently the most commonly performed bariatric operation worldwide for the treatment of morbid obesity. However, concerns have been raised about its outcomes in terms of gastroesophageal reflux disease (GERD) [1]. Additionally, the incidence of hiatal hernia (HH) in patients with obesity ranges from 23 to 52.6% and is considered an independent risk factor for the development of GERD [2, 3]. Daes et al. found that approximately 25% of patients undergoing LSG had HH [4]. Many surgeons believe that concurrent hiatal hernia repair (HHR) should be performed during LSG to reduce the risk of postoperative reflux [5–7]. The International Sleeve Gastrectomy Expert Panel Consensus Statement advocates for the aggressive identification and repair of HH during LSG [8].

On the other hand, Laparoscopic bariatric surgery is constantly advancing to minimize surgical trauma and enhance cosmetic outcomes, particularly among young women and patients who are concerned about the appearance of their scars [9]. In line with this, we developed the Bikini-Line Sleeve Gastrectomy (BLSG) approach to make the port site scars from LSG less noticeable. This approach involves placing the trocars at the lower abdomen, along the curved line just above the symphysis pubis [4]. The aim of this placement is to achieve a more aesthetically pleasing outcome, as the scars are concealed within the bikini line. During the implementation of the BLSG technique, it was observed that some patients also had a concurrent hiatal hernia (HH) that needed to be addressed. The challenge was to perform the hiatal hernia repair using the same access points without compromising the safety or efficiency of the repair.

In this study, we present a novel approach known as “Bikini-Line Hiatal Hernia Repair” (BLHHR) for repairing hiatal hernia during BLSG. The aim of this manuscript is to describe our initial experience and evaluate the feasibility and safety of this approach.

## Patients and Methods

Between September 2020 and October 2022, we conducted a prospective preliminary study on eligible patients with obesity for BLSG at Alexandria Main University Hospital. Patients diagnosed with hiatal hernia (HH) underwent BLSG with simultaneous laparoscopic repair of the hernia through the same access, referred to as BLHHR. Exclusion criteria encompassed prior major abdominal surgery, HH exceeding 3 cm, extensive adhesions in the lower abdomen detected intraoperatively, and a body mass index (BMI) surpassing 55 kg/m^2^. We excluded large hernia size and high BMI during this initial study to ensure greater safety and technical success. Additionally, patients with distances exceeding 34 cm between the xiphoid process and the umbilicus, 37 cm between the xiphoid process and the symphysis pubis and the anterior superior iliac spine (ASIS), and 33 cm between the xiphoid process and the ASIS were also excluded. These specific distance thresholds were established to ensure optimal ergonomics during the procedure, as detailed in the “Surgical Technique” section.

All operative procedures were performed by the same surgeon (the author) using a standardized perioperative protocol and operative technique. Informed consent was obtained from all participants, and the study received approval from the Institutional Review Board (IRB).

We collected and analyzed the demographic and anthropometric characteristics of the patients, preoperative symptoms of GERD, presence of hiatal hernia (HH), and the incidence of associated comorbidities. The surgical duration, length of hospital stay, the occurrence of operative complications, and postoperative pain scores were documented.

All participants underwent upper gastrointestinal endoscopy (UGIE) before and 6 months following surgery. Postoperative weight loss was expressed as a percentage of excess weight loss (%EWL), and changes in GERD symptoms were evaluated using a simplified clinical classification system: Grade 0 (none), Grade 1 (mild symptoms, no proton pump inhibitor (PPI) use), Grade 2 (moderate symptoms, occasional PPI use), and Grade 3 (severe symptoms, frequent PPI use) [10]. Patient scar satisfaction was assessed using four validated subscales of the Scar Assessment Questionnaire (PSAQ): appearance, consciousness, satisfaction with appearance, and satisfaction with symptoms; every subscale comprises a collection of items that elicit 4-point categorical responses, ranging from 1 to 4 points. The scoring system allocates 1 point to the most favorable category and 4 points to the least favorable category. Patients were also asked to rate their overall satisfaction with the scar appearance as very satisfied, just satisfied, or dissatisfied [4, 11]. All patients were followed up for a minimum of 6 months.

Complete diabetes (T2DM) remission was defined as fasting blood glucose (FBG) < 100 mg/dL and/or HbA1c < 6%, and partial remission was defined as FBG < 126 mg/dL and/or HbA1c < 6.5%, with both conditions met while being off anti-diabetic medication [12]. Hypertension was defined as systolic blood pressure ≥ 140 mm Hg or diastolic blood pressure ≥ 90 mm Hg [12]. The resolution of hypertension was defined as the discontinuation of all antihypertensive medications, while the improvement of hypertension was defined as a decrease in the dose or number of antihypertensive medications [13].

### Surgical Technique

**Fig. 1:**
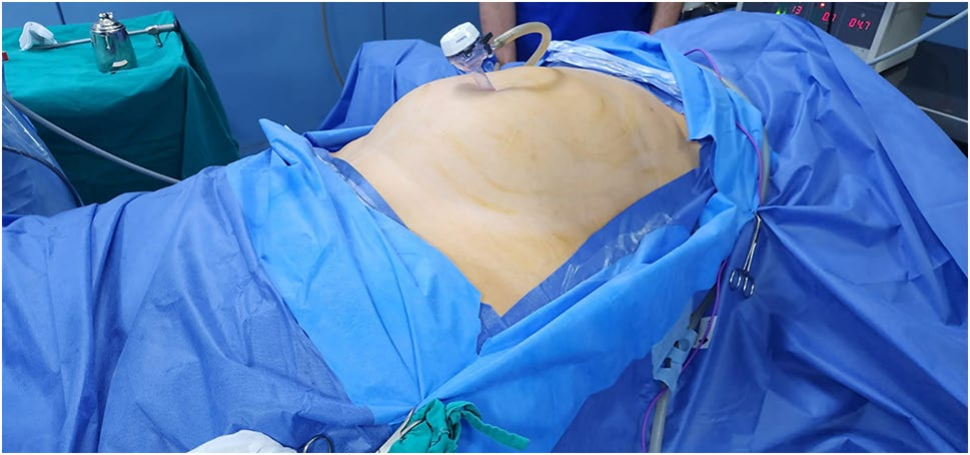
Patient position and drapes at a lower level

**Fig. 2:**
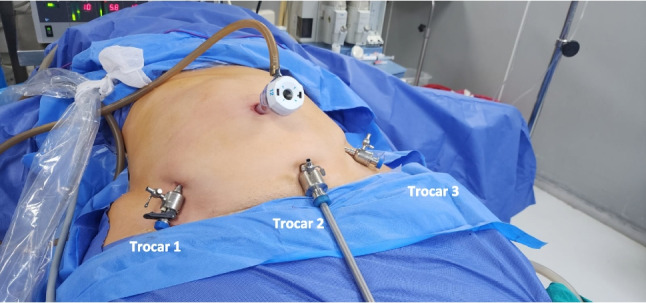
Port sites: three trocars at the bikini line and one at the umbilicus

We carried out the operative steps described previously for BLSG [4]. Patients were positioned in a modified split-leg position, with a smaller angle of splitting and the left leg was straight compared to the right leg. This positioning allowed the surgeon to have more space during suturing. The straps and draping were lowered and placed at the level of the upper one-third of the thigh to expose the lower abdomen (Fig. 1). An optical trocar was then introduced through the umbilicus to establish a closed pneumoperitoneum. Initial exploration was performed to check for major adhesions in the lower abdomen. Under visual guidance, three trocars were then inserted along the bikini line, which is the curved line just above the symphysis pubis (Fig. 2). The operating table was tilted 45° in the reverse Trendelenburg position.

**Fig. 3:**
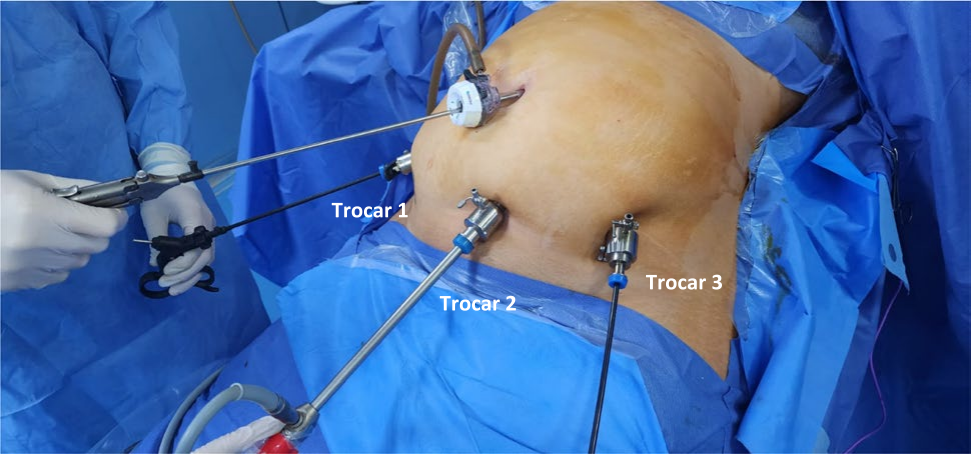
Surgeon standing on the right side of the patient. Surgeon’s two working hands: umbilical and trocar 1

A thorough inspection of the gastroesophageal junction (GEJ) and diaphragmatic hiatus was conducted to identify the presence of a HH. The dissection of the gastric greater curvature extended up to the cardio-esophageal junction. Complete mobilization of the gastric fundus involved dissecting the fat pad to expose the GEJ and identify any hidden sliding hiatal hernia. Visible signs such as a diaphragmatic defect, GEJ positioned above the diaphragm, or pericardial fat retracted into the hiatus (appearing as a dimple) indicated the presence of a hiatal hernia [2]. If a hiatal hernia was found, a complete hiatal repair was performed before gastric stapling (Fig. 3). This repair procedure included placing a traction tape around the esophagus, fully dissecting the diaphragmatic crura to the mediastinum, and reducing the gastric herniation into the abdomen, ensuring at least 3 cm of the intra-abdominal esophagus [14]. The hiatal crural defect was posteriorly repaired using two or more interrupted 2/0 Ethibond sutures (Ethicon, Johnson & Johnson, Somerville, NJ), and additional anterior sutures were applied to approximate the crura anteriorly. Mesh was not used in any patient, and a 40-French calibration tube was inserted to test the repair. After the hiatal repair, the dissection of the gastric greater curvature, stapling, suturing, and extraction of the resected stomach was performed as previously described for BLSG [4]. All incisions were closed using absorbable monofilament 3/0 Monocryl sutures (Ethicon, Johnson & Johnson, Somerville, NJ).

### Ergometry

To address the challenge of performing BLHHR when the ports are positioned at a distance from the GEJ and diaphragmatic hiatus, we employed laparoscopic instruments and equipment that could accommodate varying anthropometric measurements of patients. Reusable laparoscopic bariatric instruments, such as a 43 cm grasper and needle driver, a 42 cm camera telescope 45®, an endoscopic stapler, and a 44 cm bipolar energy source, were utilized. These instruments allowed for adequate reach and maneuverability during the procedure.

To ensure optimal ergonomics during the procedure, we established specific distance thresholds. The distance from the xiphoid to the umbilicus was set at 34 cm, ensuring that the endo-stapler and bipolar energy source could reach the diaphragmatic hiatus while leaving a 10-cm segment of the instruments accessible for manipulation outside the body. The distance from the xiphoid to the symphysis pubis was set at 37 cm to provide an optimal view with the camera telescope positioned 10 cm away from the target point, as per recommendations [15]. Lastly, the distance from the xiphoid to the anterior superior iliac spine was set at 33 cm to accommodate the length of the graspers and needle drivers, allowing 10 cm of the instruments to extend outside the body.

**Fig. 4:**
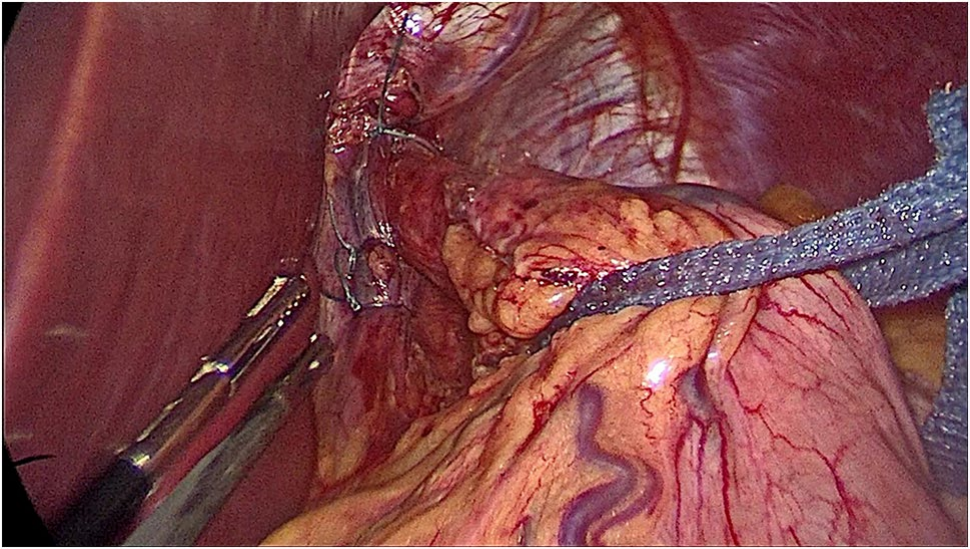
Hiatus after full crural repair

During the inspection and exploration of the diaphragmatic area of the hiatus, the surgeon stood on the patient’s right side, while the cameraman was positioned between the patient’s legs. The assistant, on the other hand, stood on the left side of the patient. The camera telescope was inserted through trocar 2, and the surgeon’s two working trocars were trocar 1 and the umbilical trocar, as depicted in Fig. 4. This positioning and trocar placement allowed for optimal visualization and accessibility during the procedure.

It is noteworthy to mention that the main challenge we encountered at the beginning of this study was achieving good access to the GEJ. To enhance the safety and effectiveness of BLHHR, while preserving the desired aesthetic benefits, it would be crucial to optimize ergonomics and equipment selection based on individual patient anthropometric measurements. Additionally, adjusting the operating table, trocar placement, and laparoscopic instruments to accommodate the Bikini line incision would be necessary to provide optimal visualization and access to the gastroesophageal junction (GEJ). One important ergonomic technique is to position the camera lens between the surgeon’s instruments while taking suture bites. However, during knot tying, the camera lens is withdrawn and rotated to create more space for throwing the knot. This entire process is facilitated by the proper elevation of the esophagus, which is achieved using a traction tape handled by the second assistant.

### Postoperative

Patients were allowed clear fluids on postoperative day 1. Standard intravenous analgesia (non-steroidal anti-inflammatory; ketorolac tromethamine 30 mg as a single dose and as needed thereafter), was administered to all patients for pain control. The majority of patients were discharged home by the second postoperative day. Follow-up visits were scheduled at 10 days, 1 month, 3 months, and 6 months after surgery at the surgical obesity clinic. During these visits, patients were monitored for complications, satisfaction with the appearance of the port site scars, changes in GERD symptoms, resolution of associated comorbidities, and postoperative weight loss.

### Statistical Analysis

Data were analyzed using IBM SPSS software package, version 18 (IBM Company, Chicago, IL, USA). Results were reported as mean ± standard deviation for continuous variables or as percentages for categorical variables. Comparison of means for continuous variables was done using *t* tests while the comparison of categorical variables was done using chi-squared tests or Fisher’s exact *t* test when appropriate. A *p* value of < 0.05 was considered significant.

## Results

During the study period, a total of 891 patients were eligible for the study and formed our cohort. Hiatal hernia was diagnosed in 89 (9.9%) of these patients and they underwent BLSG with concomitant hiatal hernia repair (BLHHR), forming group A. The remaining 802 patients comprised group B and underwent only BLSG. The two groups were comparable concerning age, female sex incidence, BMI, and associated comorbidities. Table 1 presents the demographic characteristics, anthropometric measurements, and prevalence of comorbidities for all participants. Hiatal hernia incidence and changes in GERD following surgery are demonstrated in Table 2a and b

**Table 1:** Baseline characteristics and prevalence of comorbidities

Total (*n* = 891)	BLSG+BLHHR (Group A) (*n* = 89)	BLSG (only) (Group B) (*n* = 802)	*P* value
Age (years) (range)	40.1 ± 13 (28-62)	38.9 ± 11 (26-59)	0.12
Women *n* (%)	73 (82%)	648 (80.7%)	
Weight (kg) (range)	114.5 ± 15 (92–145)	111.2 ± 13 (90–140)	0.27
BMI (kg/m^2^) (range)	43.7 ± 5.1 (37–55)	44.1 ± 5.3 (36–51)	0.43
Comorbidities (*n* (%))			
Hypertension	11(12.3%)	96 (11.9%)	0.79
T2DM	21 (23.6%)	168 (20.9%)	0.84
Sleep apnea	4 (4.5%)	45 (5.6%)	0.68
Chronic joint pain	11 (12.3%)	110 (13.7%)	0.70

Data is expressed as a percentage (%) or mean ± standard deviation

*BLSG* bikini line sleeve gastrectomy, *BLHHR* bikini line hiatal hernia repair, *BMI* body mass index, *kg* kilogram, *n* = number, *p* = significance

**Table 2:** **A** Changes in gastroesophageal reflux disease (GERD) symptoms and **B** upper gastrointestinal endoscopy (UGIE) findings

A		
	Group A (BLHHR) (*n* = 89)	Group B (BLSG) (*n* = 802)
Asymptomatic	54 (60.7%)	481 (59.9%)
Preoperative GERD symptoms	35 (39.3%)	321 (40.0%)
Grading of symptoms		
Grade 1	15 (42.8%)	138 (42.9%)
Grade 2	17 (48.6%)	154 (47.9%)
Grade 3	3 (8.6%)	39 (9.0%)
GERD symptoms remission	22 (62.8%)	141 (43.9%)* *p =* 0.02
GERD symptoms persistence	13 (37.1%)	180 (56.0%)* *p* = 0.02
De novo GERD symptoms	3 (5.5%)	39 (8.1%)
B		
	Group A (BLHHR) (*n* = 89)	Group B (BLSG) (*n* = 802)
Preoperative UGIE		
Demonstration of hiatal hernia	35/89 (39.3%)	None* *p* < 0.001
Esophagitis	10/89 (11.2%)	87/802 (10.84%)
In GERD patients	6/35 (17.1%)	54/321 (16.8%)
In asymptomatic	4/54 (7.4%)	33/481 (6.8%)
Postoperative UGIE		
Hernia recurrence	No	No
Esophagitis resolution	8/10 (80%)	66/87 (75.8%)
Resolution in GERD	6/6 (100%)	54/54 (100 %)
Resolution in asymptomatic	2/4 (50%)	12/33 (36.3%)* *p* = 0.02

Preoperative and 6 months following surgery

*N* number

*p < 0.05

### Measurements and Operative Findings

The mean distances between the xiphoid process to the umbilicus, symphysis pubis, and anterior superior iliac spine (ASIS), in all the participants, were 28.8 ± 2.2 (21–34), 33.9 ± 3.1 (32–37) and 31.2 ± 1.8 (27–33) cm, respectively. Scars from previous lower abdominal surgery were noted in 134 (15.03%) of all participants; these included scars of 102 Caesarian sections, 19 abdominoplasties, and 13 appendectomies. Exploration of the GEJ and the diaphragmatic hiatus diagnosed HH in 54 of the 89 (60.7%). HH repair and sleeve gastrectomy were uneventful. We did not have to relocate the ports; however, in two of the patients in group A and 16 of the patients in group B, a 5-mm trocar was added in the epigastrium for liver retraction.

### Postoperative Period

The mean duration of surgery, length of hospital stay, operative complications, and postoperative pain score are shown in Table 3. We had no mortality or major operative complications. Laparoscopic re-intervention was required in one patient in group B, for bleeding, and revealed a peri-gastric hematoma with no active bleeder.

**Table 3:** Mean operative time, hospital stay, complications, and pain score

	Group A (*n* = 89)	Group B (*n* = 802)
Mean operative time (min)	76.5 ± 11 (range 50–90)	58 ± 10 *p = 0.01 (range 50–70)
Mean hospital stay (days)	1.1 ± 0.3 (range 1–3)	1.1 ± 0.6 (range 1–4)
Bleeding *n* (%)	1 (1.1%)	11 (1.4%)
Superficial surgical site infection	4 (4.5%)	32 (3.9)
Portal vein thrombosis	0	2 (0.25)
Postoperative pain score (VAS)	2.9 ± 0.8 (range 2–4)	2.7±o.9 *p* = 0.81 (range 2–4)

*VAS* visual analog scale, *n* number

The percent sign (%) denotes percentage

*p < 0.05

### Cosmetic Satisfaction

The satisfaction of patients with their port-site scars, as assessed with the PSAQ at 10 days, 3 months, and 6 months, and their overall satisfaction with scar appearance, are summarized in Tables 4 and 5. No significant difference was observed between both groups.

**Table 4:** Patients’ scar assessment questionnaire (PSAQ): mean score results

	Min scores	Max scores	10 days	3 months	6 months
Group A	27	106	41.38 ± 4.4	32.12 ± 4.5	27.34 ± 4.1
Group B	32	112	43.40 ± 3.9	35.11 ± 4.0	28.20 ± 3.7

*Min* minimum (minimum score = best)

**Table 5:** The overall satisfaction with scar appearance in both groups of patients

	10 days		1 month		3 months		6 months		12 months	
	Group A	Group B	Group A	Group B	Group A	Group B	Group A	Group B	Group A	Group B
	*n* = 89 (100%)	*n* = 802 (100%)	*n* = −84 (94.3%)	*n* = 762 (95.0%)	*n* = 79 (88.7%)	*n*=749 (93.4%)	*n*=89 (100%)	*n*=80 (100%)	*n* = 16/28 (57.1%)	*n* = 182/380 (47.8%)
Very satisfied	85 (95.5%)	770 (96%)	80 (95.2%)	708 (92.2%)	71 (89.8%)	668 (89.2%)	87 (97.8%)	785 (97.9%)	14 (87.5%)	175 (96.1%)
Just satisfied	4 (4.5%)	32 (3.9%)	4 (4.7%)	54 (7%)	8 (10.1%)	8 (10.8%)	2 (2.2%)	17 (2.1%)	2 (12.5%)	7 (3.8%)

*N* number

### Weight Loss and Resolution of Comorbidities

Postoperative weight loss and resolution or improvement of associated comorbidities, are presented in Table 6.

**Table 6:** Postoperative weight loss, and resolution or improvement of comorbidities

Postoperative follow-up visits		%EWL	T2DM A (*n* = 21) B (*n* = 168)	Hypertension A (*n* = 11) B (*n* = 96)	Joint pain A (*n* = 11) B (*n* = 110)	OSA A (*n* = 4) B (*n*= 45)
1 month	Group A		16 (76.2%)	5 (45.4%)		
	Group B		130 (77.3%)	46 (47.9%)		
3-month	Group A	38.4 ± 6.1%	17 (80.9%)			
	Group B	37.2 ± 4.1%	146 (86.9%)			
6-month	Group A	53.3 ± 13.7%	18 (85.7%)	6 (54.5%)	6 (54.5%)	3 (75%)
	Group B	51.7 ± 11.2%	148 (88.1%)	54 (56.2%)	68 (61.8%)	30 (66.5%)
12-month	Group A	57.8 ± 6.7%	(100%)	7 (63.6%)		100%
	Group B	56.9 ± 4.9%		60 (62.5%)		

*%EWL* percentage excess weight loss, *T2DM*= type 2 diabetes, *A* group A, *B* group B. *N* number

All participants were followed up for a minimum of six months and attended the follow-up visits at 10 days and 6 months. The follow-up rate at 1, 3 and 12 months are presented in Tables 4 and 5. Twenty-eight patients in Group A and 380 patients in Group B completed 12 months of follow-up after surgery. Out of these, 16 (57.14%) and 182 (47.89%), respectively, attended the 12-month follow-up visit. The overall follow-up rate for both groups was 83.4% and 78%, respectively.

## Discussion

The reported rate of combined laparoscopic sleeve gastrectomy (LSG) and hiatal hernia repair (HHR) ranges from 14 to 24% [16, 17]. However, in our current study involving 891 patients who underwent BLSG, 9.9% of them were diagnosed with a hiatal hernia (HH). These patients underwent BLSG along with concomitant hiatal hernia repair (HHR), which we have named “Bikini-Line Hiatal Hernia Repair” (BLHHR) (group A). Previous studies have demonstrated that performing hiatal hernia repair during sleeve gastrectomy is relatively safe and does not carry an increased risk of complications [17]. Nonetheless, we had concerns regarding the potential technical challenges we might face when performing BLHHR using the proposed access through the lower abdomen. The primary challenge of this approach was primarily attributed to the placement of trocars at a distance from the GEJ and the hiatal region of the diaphragm, as well as the potential limitations in achieving optimal instrument triangulation.

In this study, the mean distances between the xiphoid process to the umbilicus, symphysis pubis, and anterior superior iliac spine (ASIS) in all participants were appropriate for the instruments used and maintained an acceptable spacing between trocars, ensuring satisfactory instrument triangulation and ergonomics (Figs. 2 and 4). Moreover, the scars from the previous lower abdominal surgery in 15.6% of all participants did not prevent the lower placement of trocars at the bikini line. The utilization of long instruments, endo staplers, and a 45° camera lens telescope facilitated convenient exposure and clear visualization of the GEJ, the left crus, and the diaphragmatic hiatus, without compromising ergonomic considerations during hiatal dissection and hernia repair. Furthermore, the access approach employed during BLHHR enabled the performance of gastric greater curvature dissection, fundus mobilization, and gastric stapling, similar to the standard LSG approach.

During our study, hiatal hernia was diagnosed preoperatively with UGIE in 39.3% of patients, while in the remaining 54 (60.7%) patients, it was identified during surgery. Preoperative diagnosis of hiatal hernia is often challenging, even in high-volume centers, and exhibits low diagnostic sensitivity. Particularly, the detection of small hiatal hernias during preoperative endoscopy can be challenging, unlike during surgery, where direct visualization of the gastrointestinal anatomy is achievable [14]. Intraoperative identification of hiatal hernia (HH) is considered the gold standard; however, pinpointing a small HH in an obese population can be arduous [2, 18]. The diagnosis of hiatus hernia (HH) during UGIE may be impaired by the subjective and indirect evaluation of the location of the LES and the crural diaphragm. This impairment could explain the occasional discrepancy in results observed in certain studies. However, preoperative HH diagnosis could be enhanced by the use of high-resolution manometry (HRM), especially when considering anti-reflux surgery. HRM allows for an accurate diagnosis of HH and provides a better classification compared to endoscopy and radiology. It enables clear identification of the crural diaphragm and LES, as well as the evaluation of their anatomical relationship [18].

The baseline characteristics and prevalence of associated comorbidities were not significantly different between both patient groups. Approximately one-third of patients in each group experienced preoperative GERD symptoms, primarily falling into Grade I and II categories. Furthermore, preoperative endoscopy findings revealed the presence of esophagitis in both groups, irrespective of symptomatology, with a higher incidence among symptomatic patients.

The mean duration of surgery for group A patients was 76.5 ± 11 min, which was significantly longer compared to the 58 ± 10 min observed for those who underwent only BLSG (*p* = 0.01). There was an increase in operative time of approximately 18 min in BLHHR patients. However, both the operative time and hospital stay remained within the accepted range for these procedures.

We observed no instances of mortality or postoperative leak complications. Bleeding occurred in 12 participants (1.34% of the total), with no significant difference in the incidence rate between the two patient groups (1.1% and 1.4%, respectively). Conservative management was effective in treating bleeding, and laparoscopic re-intervention was required in only one patient. Previous studies have also reported no increase in morbidity, length of stay, or complication rates following LSG with concomitant HHR [6, 7, 14, 19]. According to the International Federation for the Surgery of Obesity and Metabolic Disorders (IFSO), the incidence of postoperative complications for LSG is 2.12%, and the mortality rate varies between 0.18 and 0.27% [20]. Bleeding is the most frequent complication after LSG, occurring in 1.16– 4.94% of cases [21]. Superficial wound infection was observed in 4% of the participants and responded well to wound dressing and antibiotic treatment.

The mean postoperative pain score following BLHHR on the Visual Analog Scale (VAS) was minimal (2.9 ± 0.8) and comparable to the scores reported when the conventional approach was used [22]; concomitant hiatal hernia repair (HHR) did not increase the postoperative pain. This approach offers an additional advantage as lower abdominal scars are associated with less postoperative pain when compared to upper abdominal scars. Moreover, extracting the sleeve specimen through the umbilicus is even associated with lower postoperative pain [4].

Most patients in this study (89.8 to 97.8% in group A and 89.2 to 97.9% in group B) reported increased satisfaction with the appearance of their scars. The Patient Scar Assessment Questionnaire (PSAQ) results indicated a gradual improvement in scores, reaching their peak at the 6-month follow-up. Furthermore, placing the scars in the lower abdomen and below the abdominal folds reduced their visibility and increased patient satisfaction. Another potential advantage of this approach is the possibility of completely removing the scars during a future abdominoplasty. The aesthetic enhancement and the subsequent positive psychological impact unquestionably contribute to improving the patients’ quality of life [4].

The mean %EWL and comorbidity resolution at 6 months postoperative were satisfactory and comparable to the results reported following conventional LSG [4, 19, 23]. Patients who completed 12 months following surgery had successful weight loss; the mean %EWL was 57.8 ± 6.7% and 56.9 ± 4.9% in groups A and B, respectively; Dietel et al. reported a mean %EWL of 62.7% 1 year following LSG [19].

In our study, most of the comorbidities in both groups of patients improved or resolved 6 months following surgery. T2DM remission started from the first postoperative month and complete remission was achieved in 85.7% and 88.1% in group A and B patients, respectively, at six months. Our results are in line with the findings of Pham et al. [24], Leonetti et al. [25], and Pournaras et al. [26]. These studies showed remission rates ranging from 26 to 80% at various time intervals. The rapid good remission in the first postoperative month could be due to the strict all-fluid diet that patients receive during their early recovery period.

The significant early improvement in hypertension observed in our patients is consistent with the findings reported in previous studies. Samson et al. [27] observed a decrease in blood pressure within the first month, with improvement in hypertensive symptoms starting from the early postoperative days. Xiaoqiang et al. [28] reported a reduction in blood pressure within 10 days after the operation, and at 12 months, hypertension was resolved in 87% of patients and lowered in 100% of patients. Similarly, Ruiz-Tovar et al. [22] and Hutter et al. [29] found that 66.6% and 68% of their patients, respectively, achieved hypertension-resolution 1 year after LSG. These studies demonstrate that LSG can reduce blood pressure before significant weight loss occurs, indicating the potential involvement of neural and hormonal mechanisms in blood pressure reduction. Furthermore, the improvement in chronic joint pain and sleep apnea observed in our patients aligns with the results reported by Xiaoqiang et al. [28], who noted that joint pain was resolved in 78% of patients and sleep apnea syndrome was no longer present in 86% of patients within 12 months after LSG.

In the present study, 6 months after surgery, hiatus hernia repair (BLHHR) was found to be associated with a significant remission of GERD symptoms (62.8% compared to 43.9% in the BLSG-alone patients) and a lower rate of de novo symptoms (5.5% and 8.1%), respectively. These findings align with previously reported results. Soricelli et al. demonstrated a 73.3% remission of GERD symptoms in symptomatic patients without de novo symptoms, while Ruscio et al. reported 89% GERD remission [6, 30]. Moreover, a majority of studies included in a systematic review reported a similar significant resolution of GERD following sleeve gastrectomy with concurrent HHR [1, 2, 6]. The improved data observed with BLHHR over BLSG alone can be attributed to the underlying anatomical changes associated with both procedures. The LES complex, crucial for preventing GERD, is compromised during LSG. Adding HHR strengthens the anti-reflux barrier, repairing anatomical alterations in the LES and sling fibers at the cardia linked to reflux symptoms. LSG alone leads to intra-thoracic migration of the sleeved stomach due to gastric fundus loss, and the frequent phreno-esophageal ligament disruption weakens the structural framework between the intrinsic sphincter and external crura. These factors, along with increased intra-gastric pressure, contribute to GERD.

Conversely, some authors have reported no significant improvement in GERD symptoms. Samakar et al. noted that 65.4% of symptomatic patients experienced persistent reflux symptoms, and 15.6% developed de novo symptoms [31]. Santonicola et al. found that LSG with HHR did not improve GERD symptoms and led to a significantly higher heartburn intensity-frequency score compared to LSG-alone patients [14]. Additionally, Dakour et al. discovered that following LSG with concurrent HH repair, only 21.3% of symptomatic patients achieved symptom remission, while 40.4% experienced worsening symptoms and 41.4% developed de novo symptoms [10]. Moreover, postoperative endoscopy did not reveal any hernia recurrence and demonstrated that HHR led to the resolution of esophagitis in all symptomatic GERD patients and in 50% of asymptomatic patients. Dakour et al. demonstrated that esophageal symptoms were not indicative of the presence of endoscopic lesions or reflux; patients with hiatus hernia (HH) can experience GERD symptoms without evidence of esophagitis on endoscopy, can have esophagitis without symptoms, or may even have no symptoms or esophagitis at all [12].

### Limitations

This study has several limitations. Firstly, the relatively small number of patients who underwent concurrent HHR. Secondly, the majority of patients had a short follow-up period of less than 12 months. Thirdly, there is a need for better standardization of endoscopic and intraoperative evaluation of HH in terms of diagnosis and size. Lastly, there was a lack of reflux testing to assess GERD symptoms.

## Conclusion

BLHHR was found to be potentially safe, feasible, and effective. Outcomes regarding patient scar satisfaction, weight loss, improvement of associated comorbidities, and GERD symptoms, were not compromised. The aesthetic gain achieved by BLSG was maintained. It could potentially be offered to a selected group of patients who are conscious about their scar appearance. However, this was a preliminary study conducted on a relatively small number of HH patients over a short period. To assess the widespread applicability and safety of BLHHR, long-term prospective controlled studies involving a larger number of patients are needed.
